# Supplementary material for: A Bacterial Genome and Culture Collection of Gut Microbial in Weanling Piglet
Source: Microbiol Spectr. 2022 Feb 16;10(1):e02417-21. doi: 10.1128/spectrum.02417-21 (PMC8849097; doi:10.1128/spectrum.02417-21)
Supplement: SUPPLEMENTAL FILE 1 — Supplemental material. Download SPECTRUM02417-21_Supp_1_seq9.pdf, PDF file, 1.2 MB [file spectrum02417-21_supp_1_seq9.pdf]

Supplementary method

A total of 25 kind of culture media were used for bacteria culture of gut of piglets. The samples were suspended with PBS supplemented with 0.1% cysteine and serially diluted with tenfold in an anaerobic chamber (Bactron Anaerobic Chamber, Bactron IV-2, Shellab, USA). The diluted suspension was then spread on agar plates and incubated under anaerobic condition with gas flow composition of 90% N_2, 5% CO_2 and 5% H_2 at 37 °C.

The culture media used in this study were listed as below:

5% sheep blood-MPYG: MPYG+5% sterile defidrinated sheep blood

MPYG：MPYG Medium

Kana- MPYG: MPYG Medium+ Kanamycin

5% sheep blood-BHI: Brain Heart Infusion Medium +5% sterile defidrinated sheep blood

BHI：Brain Heart Infusion Medium

Kana-BHI：Brain Heart Infusion Medium+ Kanamycin

5% sheep blood-DM：DM Medium +5% sterile defidrinated sheep blood

DM：DM Medium

5% sheep blood-GMM：Gut microbiota Medium+5% sterile defidrinated sheep blood

GMM：Gut microbiota Medium

5% sheep blood-R2A：R2A Medium+5% sterile defidrinated sheep blood

R2A：R2A Medium

5% sheep blood-SCH：SCH Medium+5% sterile defidrinated sheep blood

SCH：SCH Medium

5%sheep blood-Spore：Spore Medium +5% sterile defidrinated sheep blood

Spore Medium

5% sheep blood- Columbia：Columbia Medium+5% sterile defidrinated sheep blood

Columbia Medium

Kana- Columbia：Columbia Medium+ Kanamycin

2216：DSMZ 2216 Medium

27：DSMZ 27 Medium

98：DSMZ 98 Medium

AM：AM medium

GYM：GYM Medium

NA：Nutrient Agar Medium

1. Gut microbiota Medium

Component	Amount/L
Tryptone Peptone	2 g
Yeast Extract	1 g
D-glucose	0.4 g
L-cysteine	0.5 g
Cellobiose	1 g
Maltose	1 g
Fructose	1 g
Meat Extract	5 g
KH_2PO_4	100 mL
$MgSO_4 \cdot 7H_2O$	0.002 g
$NaHCO_3$	0.4 g
$NaCl_2$	0.08 g

$CaCl_2$	1 mL
Vitamin K (menadine)	1 mL
$FeSO_4$	1 mL
Histidine Hematin Solution	1 mL
Tween 80	2 mL
ATCC Vitamin Mix	10 mL
ATCC Trace Mineral Mix	10 mL
Acetic acid	1.7 mL
Isovaleric acid	0.1 mL
Propionnic acid	2 mL
Butyric acid	2 mL
Resazurin	4 mL
Noble Agar	12 g
pH	7.2

2. *Spore Medium*

Component	Amount/L
Yeast Extract	1 g
Beef extract	1 g
Tryptone Peptone	2 g
Glucose	10 g
$FeSO_4$	0.001 g
Distilled water	1000 mL
Noble Agar	15 g
pH	7.2

3. *DM Medium*

Solution A:

Component	Amount/L
K_2HPO_4	0.5 g
NH_4Cl	1.0 g

Na_2SO_4	1.0 g
$CaCl_2 \cdot 2H_2O$	0.1 g
$MgSO_4 \cdot 7H_2O$	2.0 g
Na-DL-lactate	2.0 g
Yeast extract	1.0 g
Na-resazurin solution (0.1% w/v)	0.5 mL
Distilled water	980.0 mL

Solution B:

Component	Amount/L
$FeSO_4 \cdot 7H_2O$	0.5 g
Distilled water	10 mL

Solution C:

Component	Amount/L
Na-thioglycolate	0.1 g
Ascorbic acid	0.1 g
Distilled water	10 mL

The solution B and solution C are added after the solution A has been boiled, adjusted pH to 7.8 with NaOH, Fixed capacity to 1000 mL, enter nitrogen to remove oxygen, Autoclave 15 min at 121℃.

4. *R2A Medium*

Component	Amount/L
Yeast extract	0.5 g
Peptone	0.5 g
Casein hydrolysate	0.5 g
Glucose	0.5 g
Starch, soluble	0.5 g
K_2HPO_4	0.3 g

$MgSO_4$	0.024 g
α-Ketopropionic acid sodium salt	0.3 g
Noble Agar	15.0 g
Distilled water	1000 mL
pH	7.2 ± 0.2

5. *Brain Heart Infusion Medium*

Component	Amount/L
Dehydration Brain infusion powder	12.50 g
Dehydration Beef Heart Infusio	1.50 g
Proteose Peptone	10.00 g
Glucose	2.00 g
NaCl	5.00 g
$NaHPO_4$	2.50 g
Noble Agar	15.00 g
pH	7.4

6. *Columbia Medium*

Component	Amount/L
Casein Tryptone	10.0 g
Pepsin Hydrolytes	5.0 g
Heart Pancreatin Hydrolytes	3.0 g
Yeast Extract	5.0 g
Corn starch	1.0 g
NaCl	5.0 g
Noble Agar	15.0 g
Distilled water	1000 mL
pH	7.3 ± 0.2

7. *GYM Medium*

Component	Amount/L
Glucose	4.0 g

Yeast Extract	4.0 g
Malt Extract	10.0 g
$CaCO_3$	2.0 g
Noble Agar	12.0 g
Distilled water	1000 mL
pH	7.2

8. *AM medium*

Component	Amount/L
KH_2PO_4	0.10 g
$(NH_4)_2SO_4$	0.25 g
$CaCl_2 \cdot 2H_2O$	0.05 g
$MgSO_4 \cdot 7H_2O$	0.02 g
Trace elements	1.0 mL
Yeast extract	0.1 g
Na gluconate	0.5 g
Distilled water	1000 mL
Noble Agar	15.0 g
pH	5.0 - 5.5

9. *2216 Medium*

Component	Amount/L
Peptone	5.0 g
Yeast Extract	1.0 g
Ferric Citrate	0.1 g
NaCl	19.45 g
$MgCl_2$	8.8 g
Na_2SO_4	3.24 g
$CaCl_2$	1.8 g
KCl	0.55 g
$NaHCO_3$	0.16 g

KBr	0.08 g
$SrCl_2$	34.0 mg
H_3BO_3	22.0 mg
$Na_2O \cdot nSiO_2$	4.0 mg
NaF	2.4 mg
NH_4NO_3	1.6 mg
Na_2HPO_4	8.0 mg
Noble Agar	15.0 g
pH	7.6 ± 0.2

10. 98 Medium

Component	Amount/L
Yeast extract	1.0 g
Mannitol	10.0 g
Noble Agar	15.0 g
Soil extract	200 mL
Distilled water	800 mL
Noble Agar	15.0 g
pH	7.2

Soil extract:

Component	Amount/L
Air-dried garden soil	80.0 g
Na_2CO_3	0.2 g
Distilled water	200 mL

11. 27 Medium

Component	Amount/L
Yeast extract	0.3 g
Na_2-succinate	1.0 g
(NH_4)-acetate	0.5 g

Fe(III) citrate solution (0.1% in H_2O)	5 mL
KH_2PO_4	0.5 g
$MgSO_4 \cdot 7H_2O$	0.4 g
NaCl	0.4 g
NH_4Cl	0.4 g
$CaCl_2 \cdot 2H_2O$	0.05 g
Vitamin B12 Solution (10 mg in 100 mL H_2O)	0.40 mL
Trace element solution SL-6 (See below)	1.00 mL
L-Cysteine hydrochloride	0.3 g
Resazurin (0.1%)	0.5 mL
Distilled water	1000 mL
Noble Agar	15.0 g
pH	6.8

12. *Nutrient Agar Medium*

Component	Amount/L
Peptone	5.0 g
beef extract	30.0 g
NaCl	5.0 g
Distilled water	1000 mL
Noble Agar	15.0 g
pH	7.0 - 7.2

13. *SCH Medium*

Component	Amount/L
Tryptone Peptone	8.2 g
Peptone	2.5 g
Peptone from soya	1.0 g
Glucose	5.8 g
Yeast extract	5.0 g
NaCl	1.7 g

$NaHCO_3$	0.8 g
Cysteine-HCl·H_2O	0.4 g
Haemin	0.01 g
Tris	15.0 g
Distilled water	1000 mL
Noble Agar	15.0 g
pH	7.2

14.MPYG Medium

Component	Amount/L
Tryptone Peptone	5.0 g
Peptone	3.0 g
Peptone from soya	2.0 g
Polypeptone	1.0 g
Casein	1.0 g
Yeast Extract	10.0 g
Beef extract	5.0 g
Glucose	5.0 g
K_2HPO_4	2.0 g
Tween 80	0.5 mL
Maltose	0.5 g
Cellobiose	0.5 g
Starch, soluble	0.5 g
Glycerol	0.5 mL
Cysteine-HCl·H_2O	0.5 g
$Na_2S \cdot 9H_2O$	0.25 g
Resazurin	1.0 mg
Salt solution	40.0 mL
Trace elements	10.0 mL
Vitamin solution	10.0 mL
Distilled water	930 mL
Haemin solution	10.0 mL

Vitamin K1 solution	0.2 mL
Noble Agar	15.0 g
pH	7.0

The vitamin K1, Haemin solution, Trace elements, Vitamin solution and the cysteine are added after the medium has been boiled and cooled under CO_2. Adjust pH to 7.0 using NaOH. Distribute under N_2 and autoclave 15 min at 121℃.

Haemin solution:

Dissolve 50 mg Haemin in 1 mL 1 N NaOH; make up to 100 mL with distilled water. Store refrigerated.

Vitamin K1 solution:

Dissolve 0.1 mL of vitamin K1 in 20 mL 95% ethanol and filter sterilize. Store refrigerated in a brown bottle.

Supplementary Figures

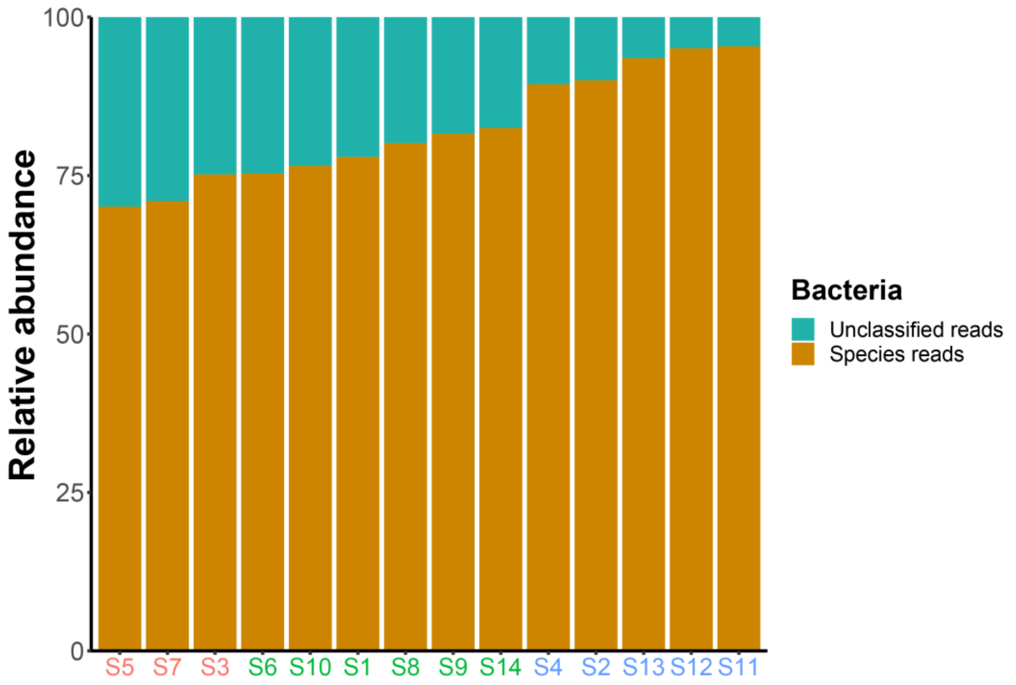

Supplementary Figure 1

Supplementary Figure 1 | The proportion of reads that were and were not classified at the species level.

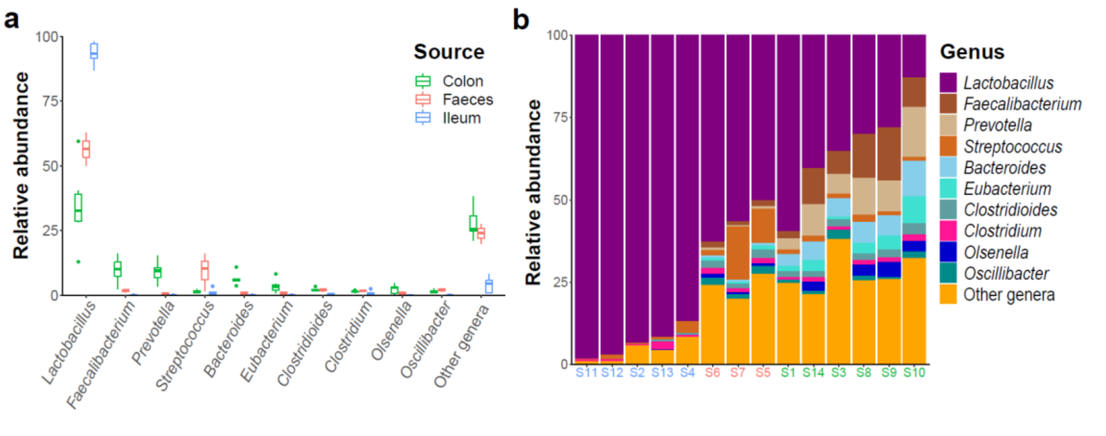

Supplementary Figure 2

Supplementary Figure 2 | a-b, The comparison of genus-level proportional abundance in the ileum, colon, and faeces of weanling piglets.

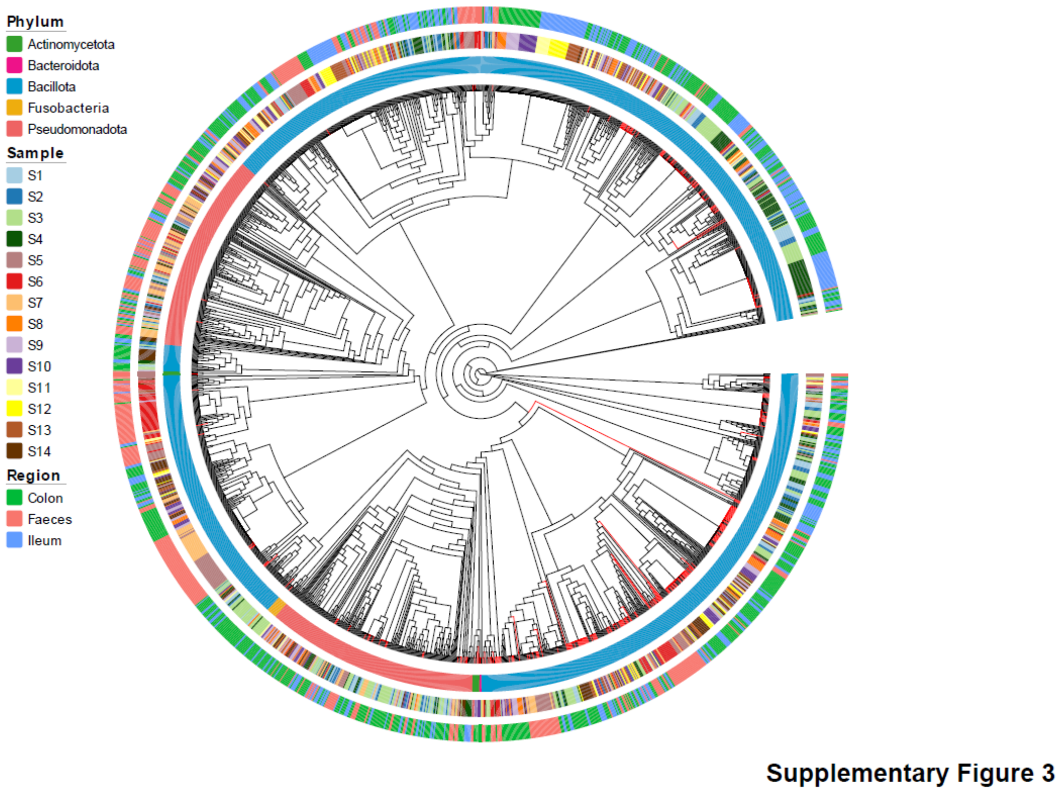

Supplementary Figure 3 | The 16S rRNA gene sequence phylogenetic tree of 1,476 strains. Novel clusters are highlighted by red clades. Phylum, sample, and region are display in the first, second, and third outer layer, respectively.

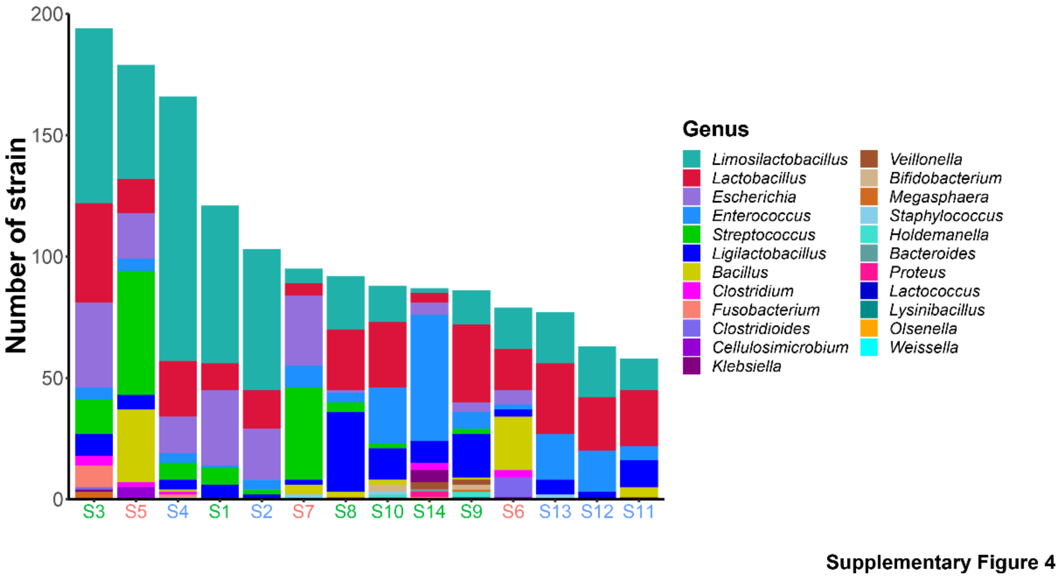

Supplementary Figure 4 | The number of cultivated bacterial strains at genus level from 14 samples.

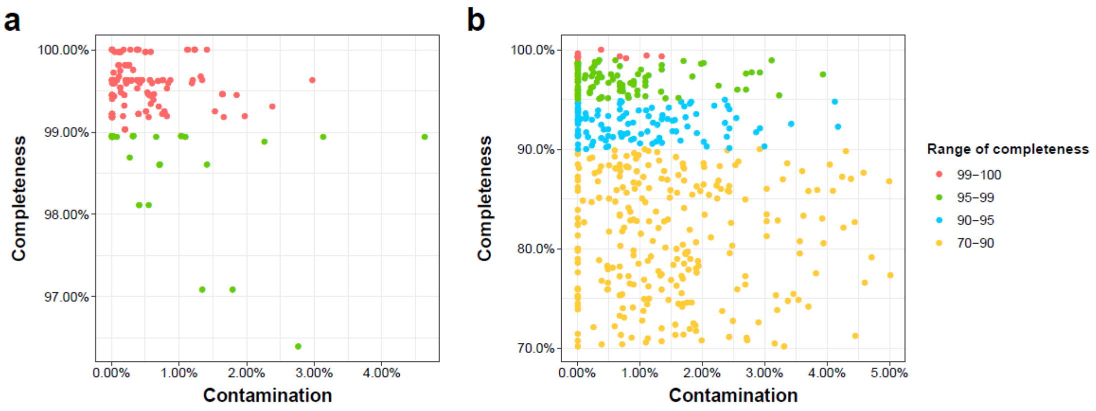

Supplementary Figure 5

Supplementary Figure 5 | Quality assessment of the genomes. a-b, Completeness and contamination of 266 isolated genomes (a) and 482 MAGs (b), respectively.

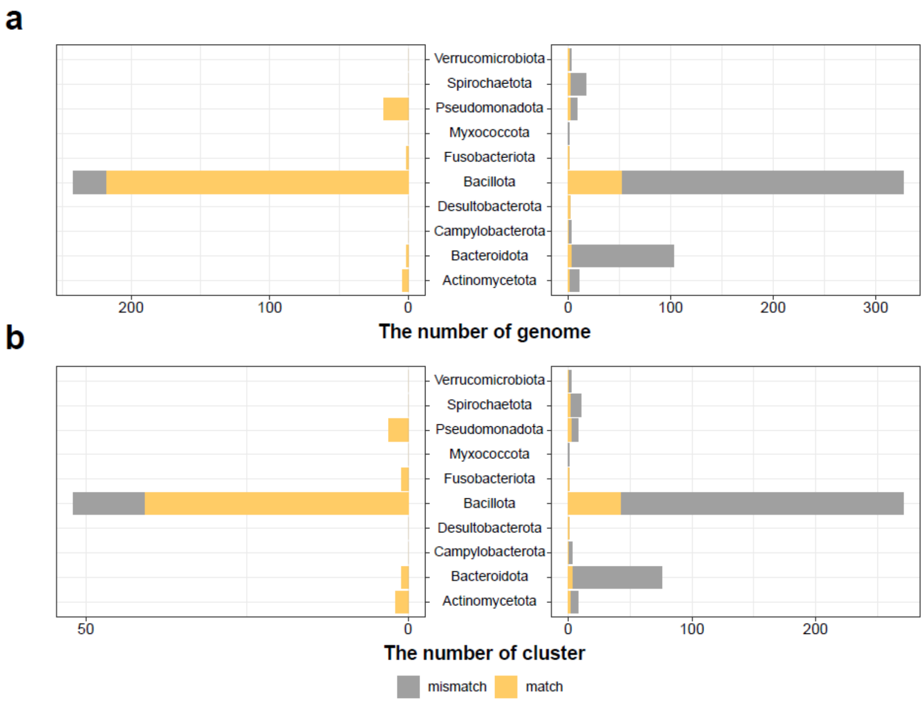

Supplementary Figure 6

Supplementary Figure 6 | The taxonomy of the 743 bacterial genomes. a-b, The distribution of isolated genomes and MAGs across different phylum. Statistics show the number of genomes (a) and clusters (b), respectively.

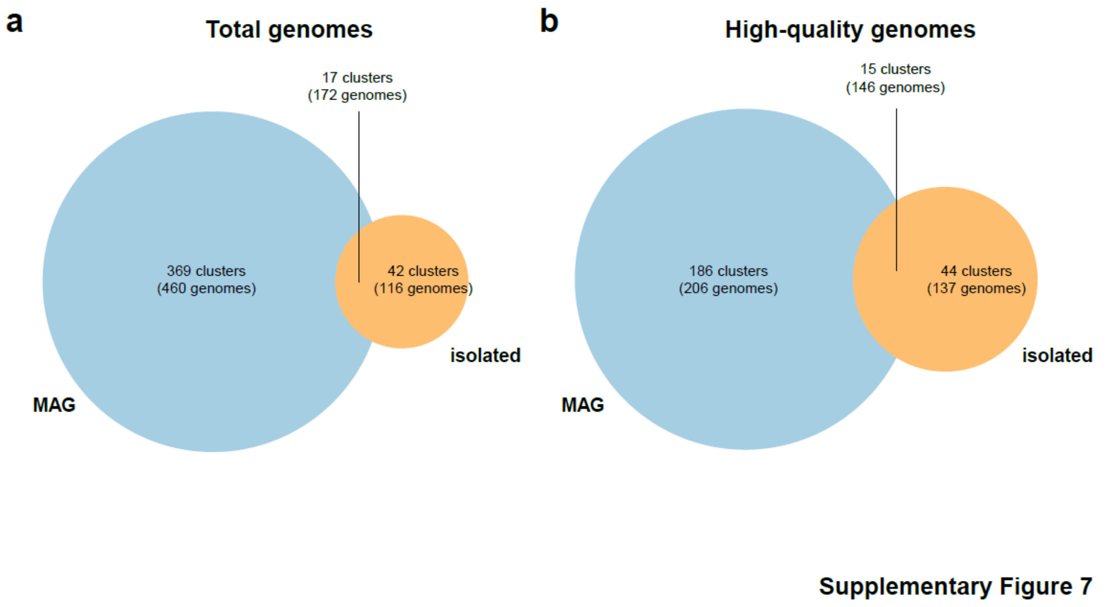

Supplementary Figure 7 | Comparison of MAGs and isolated genomes in different quality standards. a, MAGs with >70% completeness and <5% contamination, while isolated genomes with >90% completeness and <5% contamination. **b**, Both MAGs and isolated genomes are high quality (with >90% completeness and <5% contamination).

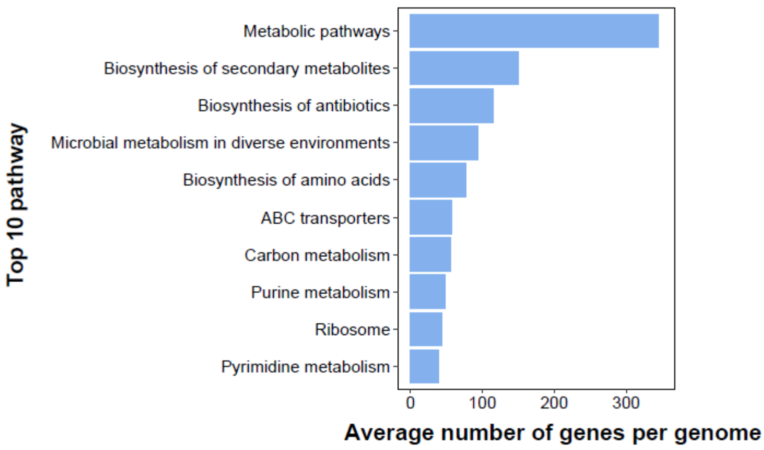

Supplementary Figure 8

Supplementary Figure 8 | The top 10 most widely distributed KEGG pathways. Sorted according to the average number of genes per genome, these 10 pathways distributed in almost all genomes.

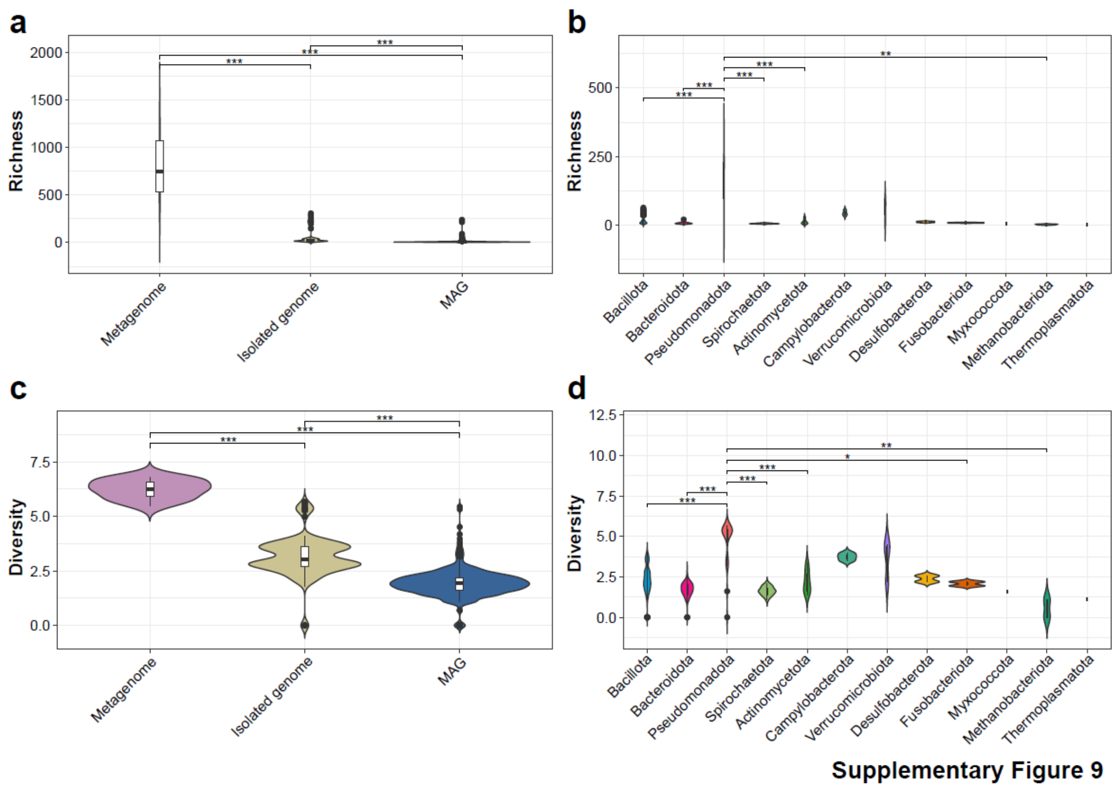

Supplementary Figure 9 | The distribution of VFs in each genome or sample. a-b, Significant differences in the richness of VFs in different methods (a) and phylum (b). **c-d**, Significant differences in the Shannon diversity of VFs in different methods (c) and phylum (d). (***$P < 0.001$, **$P < 0.01$, *$P<0.05$).

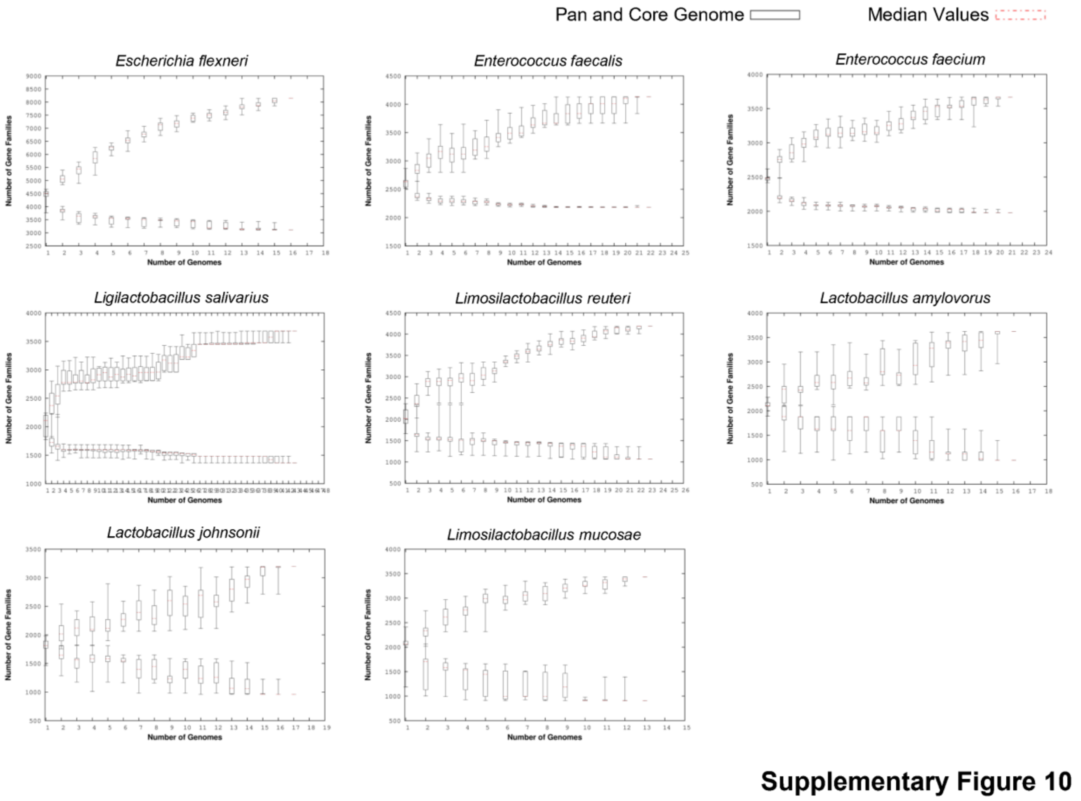

Supplementary Figure 10 | The accumulation curves of the pan and core genome of the 8 representative clusters. Box plots indicate 25th and 75th percentiles with medians shown as horizontal lines and whiskers set at 10th and 90th percentiles.

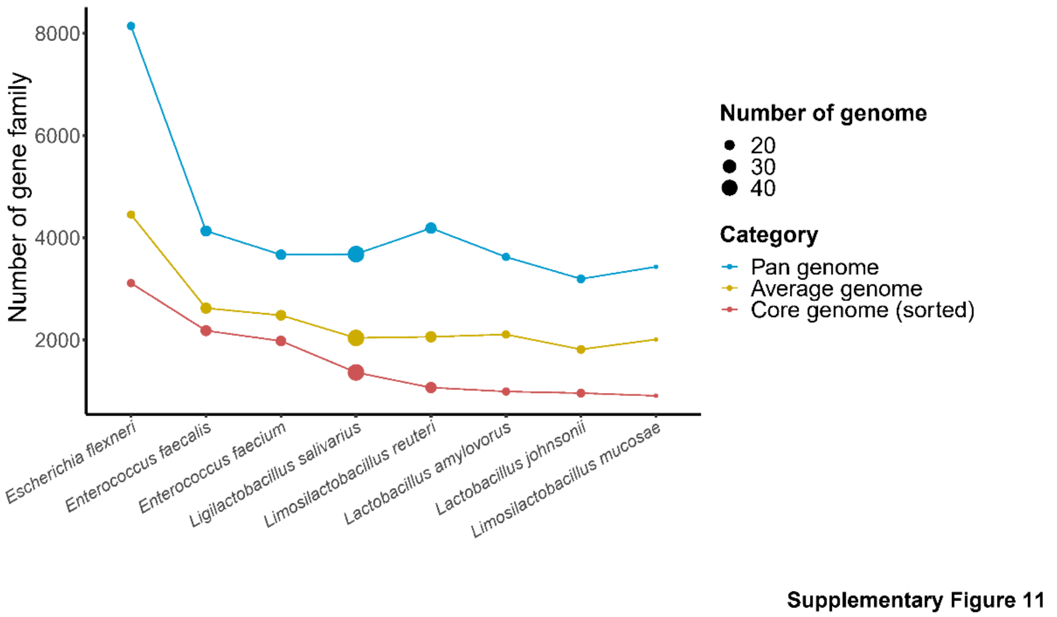

Supplementary Figure 11 | The gene family numbers of pan genome, core genome and average genome of the 8 representative clusters.
